# The Rapid Assessment of Physical Activity (RAPA) Among Older Adults

**Published:** 2006-09-15

**Authors:** Tari D Topolski, James LoGerfo, Donald L Patrick, Barbara Williams, Julie Walwick, MAJ Marsha B Patrick

**Affiliations:** Seattle Quality of Life Group, University of Washington; University of Washington School of Medicine, Seattle, Wash; University of Washington, School of Public Health and Community Medicine, Seattle, Wash; University of Washington, School of Public Health and Community Medicine, Seattle, Wash; University of Washington, School of Public Health and Community Medicine, Seattle, Wash; U.S. Army–Baylor University Graduate Program in Health and Business Administration, Waco, Tex

## Abstract

**Introduction:**

The Rapid Assessment of Physical Activity (RAPA) was developed to provide an easily administered and interpreted means of assessing levels of physical activity among adults older than 50 years.

**Methods:**

A systematic review of the literature, a survey of geriatricians, focus groups, and cognitive debriefings with older adults were conducted, and an expert panel was convened. From these procedures, a nine-item questionnaire assessing strength, flexibility, and level and intensity of physical activity was developed. Among a cohort of 115 older adults (mean age, 73.3 years; age range, 51–92 years), half of whom were regular exercisers (55%), the screening performance of three short self-report physical activity questionnaires — the RAPA, the Behavioral Risk Factor Surveillance System (BRFSS) physical activity questions, and the Patient-centered Assessment and Counseling for Exercise (PACE) — was compared with the Community Healthy Activities Model Program for Seniors (CHAMPS) as the criterion.

**Results:**

Compared with the BRFSS and the PACE, the RAPA was more positively correlated with the CHAMPS moderate caloric expenditure (r = 0.54 for RAPA, r = 0.40 for BRFSS, and r = 0.44 for PACE) and showed as good or better sensitivity (81%), positive predictive value (77%), and negative predictive value (75%) as the other tools. Specificity, sensitivity, and positive predictive value of the questions on flexibility and strength training were in the 80% range, except for specificity of flexibility questions (62%). Mean caloric expenditure per week calculated from the CHAMPS was compared between those who did and those who did not meet minimum recommendations for moderate or vigorous physical activity based on these self-report questionnaires. The RAPA outperformed the PACE and the BRFSS.

**Conclusion:**

The RAPA is an easy-to-use, valid measure of physical activity for use in clinical practice with older adults.

## Introduction

Physical activity has been demonstrated to improve management of chronic conditions and delay decline in function in older adult populations (1). Current indicators, however, show that less than 20% of U.S. adults older than 64 years engage in the surgeon general's recommended amount of physical activity, and only 11% engage in strength training (2). Additionally, several groups, including adults aged 75 years and older, women, individuals with disabilities, African Americans, and Hispanics are among the most sedentary (3).

In recent years, there has been a growing interest in a comprehensive approach to preventing and managing chronic disease that emphasizes self-management. A critical element of this self-management approach is tracking important processes and outcomes through disease registries and linking clinical practice to community-based support systems, as exemplified in the Chronic Care Model (4). (A description of this model is available from www.improvingchroniccare.org.) In our work with community support programs that complement clinical practice, including those promoting physical activity for older adults, we have found that integration of care requires common measures of key variables in both clinical settings and community support programs. We undertook this study to develop and test an easily administered questionnaire that assesses and monitors physical activity levels among older adults. Currently, there are no published reports comparing the validity of the commonly used physical activity measures with a more detailed, validated measure of actual levels of activity in older adults. In our work disseminating the EnhanceWellness Program (5), nurse coaches requested a measure that indicated more gradation of light physical activity so that they could give positive feedback as seniors evolved from being sedentary to being more active (6,7).

The goals of this study were to 1) develop a short, self-administered, and easily scored tool that could be used in a clinical setting to assess and monitor physical activity levels of older adults (aged 50 years and older), and 2) compare the accuracy of the new tool with the Patient-centered Assessment and Counseling for Exercise (PACE), a measure of level of and stage of readiness to engage in physical activity currently used by clinicians (6), and the measure of activity used in the Behavioral Risk Factor Surveillance System (BRFSS) for population-level monitoring of physical activity among adults (8) against the criterion measure Community Healthy Activities Model Program for Seniors (CHAMPS) (9-11). We chose to test the PACE because it is a measure of activity currently used for clinical counseling, and we chose the BRFSS because it is currently used for surveillance. Both tend to focus on moderate and vigorous activity, and the PACE instrument has not been validated against other measures in older populations.

## Methods

### Literature review of existing instruments

In 2000, a systematic literature review was conducted to determine whether an assessment or monitoring instrument existed that could be easily used in a primary care setting with adults aged 50 years and older. Age 50 was used because community-based organizations often use this age as the lower-end cutoff and because it was the age cutoff used in the National Blueprint program for increasing physical activity among older adults (12). Searches of Medline, PsycINFO, and the World Wide Web and queries of physical activity assessment experts and geriatric physicians helped us to identify 53 questionnaires that have been used in the past 25 years to assess physical activity. Search terms included *physical activity*, *exercise*, *questionnaire*, *instrument*, *measurement*, and *assessment*. Questionnaires were included if they were self-reported, used with adults, published or discovered through physical activity assessment experts, and available in English. These instruments were evaluated for 1) feasibility of collecting data in a primary care setting and feasibility of producing a summary for inclusion in a medical record; 2) psychometric properties of an optimal self-report screening instrument, including reliability and criterion validity; and 3) acceptability and relevance of the instrument to major ethnic populations in the United States, including Latinos and African Americans.

Members of the research team reviewed the instruments according to the following criteria: 1) dimensions of the questionnaires; 2) complexity; 3) recall time frame; 4) use as an outcome measure; 5) reliability/validity/responsiveness; 6) cultural adaptability; and 7) purpose of development. All but 12 of the 53 instruments identified in the literature search were eliminated because they were deemed to be too long and did not meet at least four of the review criteria. (A table showing questionnaires and criteria met is available from the authors). These 12 instruments were then submitted to an expert panel consisting of physical activity researchers and gerontologists who reviewed the instruments using these same criteria. The panel deemed none of these instruments to be completely acceptable either because they were too complex or because they had not been adequately validated.

### Development of the Rapid Assessment of Physical Activity instrument

Items for the Rapid Assessment of Physical Activity (RAPA) were developed based on Centers for Disease Control and Prevention (CDC) guidelines of 30 minutes or more of moderate physical activity on every or most days of the week and included additional questions added to assess strength and flexibility because of the association of these activities with preventing falls. The instrument was designed according to criteria described by Dillman (13,14) with emphasis on the cognitive burden of the questions, response layout, response format, amount of white space, font size, order of questions, repetition of the instructions, and type of examples provided. After the initial draft of the instrument was complete, the expert panel reconvened to discuss items. 

**Focus groups**

Five focus groups, with three to 12 participants in each, were conducted to assess the instrument's understandability, content, ease of completion, and cultural relevance (15). Recruitment was through a local gerontology practice at Group Health Cooperative, senior centers, and churches in the Seattle area. The focus group participants were 24% Latino, 20% Vietnamese, 26% Chinese American, 26% white, and 4% African American. Three focus groups were conducted in English, one was conducted in Spanish, and two were conducted in Vietnamese. Several versions of the newly developed instrument were presented to the focus groups for completion and discussion. All participants preferred a version of the questionnaire that included a written description and pictorial representation of the levels of physical activity (light, moderate, and vigorous), and the majority preferred a dichotomous response format. 

**Cognitive debriefing**

Cognitive debriefing is a method by which individuals assess the relevance, importance, and ease of comprehension of measures (16,17). In this step, we conducted one-on-one interviews with 12 English-speaking older adults. Participants were presented a version of the questionnaire that had been revised based on input from the focus groups. Participants were asked to think out loud as they answered the questionnaire. Upon completion of the instrument, they were asked if they thought the questions were easy to understand, whether the questions could be worded more clearly, whether the response options were appropriate and easy to understand, or if they had any other suggestions to make the instrument easier to understand and complete. The cognitive debriefing process was stopped after 12 older adults were interviewed because no new information was being elicited. Refinements to the instrument were made based on the comments of these participants and experts on physical activity and gerontology.

The final version of the RAPA (available from http://depts.washington.edu/hprc/publications/rapa.htm) was a nine-item questionnaire with the response options of yes or no to questions covering the range of levels of physical activity from sedentary to regular vigorous physical activity as well as strength training and flexibility. The instructions for completing the questionnaire provide a brief description of three levels of physical activity (light, moderate, and vigorous) with graphic and text depictions of the types of activities that fall into each category. The total score of the first seven items is from 1 to 7 points, with the respondent's score categorized into one of five levels of physical activity: 1 = sedentary, 2 = underactive, 3 = regular underactive (light activities), 4 = regular underactive, and 5 = regular active. Responses to the strength training and flexibility items are scored separately, with strength training = 1, flexibility = 2, or both = 3. Clinicians are encouraged to use this information to have a brief conversation with their patients about their current level of physical activity.

### Quantitative data collection and analysis

Participants (N = 115) for the validation segment of the study were recruited through senior centers in King County, Washington, and senior programs at Seattle Parks and Recreation. Flyers were posted at the centers, and staff at the centers announced the study during exercise and social programs. All participants in the study provided informed consent, and all procedures were approved by the institutional review board at the University of Washington.

The long-form CHAMPS (9-11) was used as the criterion self-report measure in the validation of the RAPA questionnaire because it had been validated previously against an objective measure of physical activity. The CHAMPS questionnaire was developed as a research measure and designed to give accurate estimates of caloric expenditures for all types of activity. It has been shown to be valid, reliable, and sensitive to change (10). The number of items on this questionnaire, however, makes it impractical to use in a clinical setting. The CHAMPS activities were scored as a continuous variable for determining caloric expenditure per week. To assess the discriminant validity of the three short physical activity measures, a known groups analysis compared the mean caloric expenditure between participants who did and did not meet the CDC physical activity standard of 30 minutes of moderate activity 5 days per week or 20 minutes of vigorous activity 3 days per week. This standard was used as the physical activity threshold in all analyses. Individuals met the physical activity threshold if the sum of CHAMPS moderate activities were at least 5 days per week for a total of 3 or more hours per week, or the sum of CHAMPS vigorous activities was at least 3 days per week for 1 or more hours per week. Criterion validity was assessed by calculating Spearman rank-order correlation coefficients. The known groups analysis was conducted in STATA version 8.0 (Stata Statistical Software, StataCorp, College Station, Tex) using *t* test with unequal variances. The BRFSS questions (seven items) on physical activity (8) and the PACE (6) questions were chosen to be fielded along with the CHAMPS because both can provide a summary score that equates to the physical activity threshold.

For a measure to be of value as an assessment tool, it needs to show good predictive properties. To assess the sensitivity, specificity, positive predictive value, and negative predictive value of the RAPA, the CHAMPS were scored as a dichotomous variable for defining the level of physical activity as either moderate or vigorous. Moderate-intensity activities were defined by metabolic equivalent values (METs) from 3.0 to 4.9, and vigorous-intensity activities were defined by METs of 5.0 or greater. The 2002 BRFSS questions (seven items) on physical activity (8) and the PACE (eight items) (5) were used in the construct validity analyses. The questions on both the RAPA and the PACE were scored and coded on a 5-point scale so that as the amount, frequency, and intensity of physical activity increased, the score increased (e.g., RAPA = "I almost never do any physical activities" = 1; "I do 30 minutes or more per day of moderate physical activity 5 or more days per week" = 5). The BRFSS was scored on a scale of 1 to 3, with 1 = does not engage in moderate or vigorous activities for at least 10 minutes at a time; 2 = engages in some activities, but not on a regular basis; and 3 = engages in moderate activities 5 or more days per week for at least 30 minutes per day or vigorous activities 3 or more days per week for at least 20 minutes per day.

Criterion validity of the three short physical activity measures was assessed by calculating Spearman rank-order correlation coefficients between the three physical activity measures and the CHAMPS medium caloric expenditure and total caloric expenditure. Differences in correlations were assessed using the *t* test procedure described by Blalock (18). It was expected that the RAPA would be significantly correlated with both medium and total caloric expenditure.

Readability of the instrument was assessed using the Homan–Hewitt Readability Formula because it was specifically developed for use with questionnaires (19).

Before the analysis, CHAMPS, BRFSS, PACE, and RAPA items were examined through various SPSS (SPSS, Inc, Chicago, Ill) software programs for accuracy of data entry, missing values, and fit between their distributions and the assumption of univariate analyses. No univariate outliers were found. Missing values on the number of times per week were imputed for the CHAMPS activities if values were provided for the number of hours per week. Number of times per week was imputed from the mean times per week by participants who engaged in the activity the same number of hours per week.

## Results

The sample was 72% female, 73% white, 18% African American, and 9% other race or ethnicity; the mean age (± SD) was 73.3 (± 9.6) years, and the mean body mass index (BMI) (± SD) was 27.3 kg/m^2^ (± 4.7 kg/m^2^). Compared with the 2003 American Communities Survey estimates, women and people of color are overrepresented in this sample. Because of our interest in whether the instrument could accurately identify older adults who met CDC guidelines for physical activity, we recruited through senior center exercise programs; thus, 55% of the participants met CDC criteria for being physically active, and approximately 80% engaged in some sort of physical activity program. 

Criterion validity assessments between the three physical activity measures and the CHAMPS medium caloric expenditure and total caloric expenditure are shown in Table 1. The results of the *t* test of differences in correlations showed that the RAPA was more highly correlated with CHAMPS moderate calories and total calories than either the BRFSS (*t*_102_ = 2.88, *P* < .005) or the PACE (*t*_102_ = 3.34, *P* < .001).

The results of the sensitivity, specificity, and predictive value analyses are presented in Table 2. All three short questionnaires showed good sensitivity and positive predictive value. The RAPA had the best sensitivity and negative predictive value of the three questionnaires. In this sample, information from the RAPA would lead to incorrectly classifying nonexercisers as exercisers 25% of the time. The RAPA would lead to incorrect classification of an individual as engaging in strength training 14% of the time and incorrect classification of an individual as engaging in flexibility exercises 42% of the time. A review of the data on the misclassification of individuals engaging in flexibility exercises showed that those who reported doing yoga on the CHAMPS did not indicate engaging in flexibility exercises on the RAPA, even though yoga is listed as an example. The discrepancy may be related to the fact that the RAPA specifies that the activity must be performed weekly.

The results of the discriminant known groups validity analyses are shown in Table 3. Mean caloric expenditure was calculated from the CHAMPS. For all three short physical activity measures, the group who met the physical activity standard had a significantly higher mean caloric expenditure. This indicated that all three measures were able to discriminate between those who reported inadequate and adequate moderate or vigorous physical activity. The RAPA, however, showed superior performance over the other two short measures.

Ad hoc analysis of the three physical activity questionnaires compared with the CHAMPS by respondents' BMI were performed to assess whether the RAPA correlated significantly higher with the CHAMPS than PACE or the BRFSS short physical activity questionnaires (data not shown). The RAPA correlated significantly higher with the CHAMPS for older adults whose BMI was 30 or higher, and although the correlation was higher for the group with BMI less than 30, the difference was not statistically significant. 

A Homan-Hewitt Readability Formula analysis (19) showed the readability of the RAPA to be at the sixth grade level. The observed average completion time for the RAPA was less than 2 minutes, with a range of approximately 1 to 5 minutes. 

## Discussion

Development of the RAPA included qualitative methods, cognitive debriefing with older adults, and preparation of a field trial instrument. Evaluation of the RAPA's measurement properties in this cross-sectional study is encouraging. The RAPA showed better sensitivity and negative predictive value than the other short physical activity questionnaires and better specificity and positive predictive value than the PACE. The RAPA showed good discrimination between older adults who did and did not engage in regular moderate physical activity. As is desired by nurse practitioners, the RAPA includes questions about light physical activity, a feature that allows clinicians to provide positive feedback to seniors as they move from being sedentary to being more active. Of the three short physical activity questionnaires, the RAPA is the only one that assesses strength and flexibility. It is important that a clinical physical activity measure include these areas because they are significantly related to fall reduction and maintenance of independence among older adults. 

The RAPA is readable at the sixth grade reading level and was easily understood by most participants in the study. Older adults with cognitive impairment, however, may require that the RAPA be read to them.

There are several limitations of this study: 1) all participants were volunteers recruited from Seattle-area senior centers or clinics that promote physical exercise, which may impact the generalizability of the reported results; 2) the cross-sectional data did not allow for the assessment of change over time and the value of the instrument as a monitoring tool; and 3) no observable measure of physical activity (such as an accelerometer) was used. The CHAMPS measure, however, has been shown to be sensitive to change, and the fact that the RAPA instrument tracks well with the CHAMPS provides strong criterion validity. The PACE has not been tested in such a manner.

The RAPA has been well received by geriatricians at Group Health Cooperative; many of them are using it in their clinical practice. In addition, the RAPA is being used in many research projects and program evaluations. It is being used as part of the diabetes registry in two community clinics in Seattle. As part of a quality improvement effort, the clinics are linking their patients to a community support program located at a nearby senior center, which also uses the RAPA to provide feedback to the clinics. Nurse and social work coaches involved in the EnhanceWellness program at 32 sites in seven states are also using the RAPA (5). It has been translated into Spanish and Vietnamese; however, these versions have not yet been validated. 

## Figures and Tables

**Table 1:** Correlation of the RAPA, BRFSS, and PACE With CHAMPS

	**RAPA**	**BRFSS**	**PACE**

	**Spearman Rank-order Correlation Coefficient, *r P* Value**	**Spearman Rank-order Correlation Coefficient, *r P* Value**	**Spearman Rank-order Correlation Coefficient,* r P* Value**
BRFSS	0.59<.001	—	—
PACE	0.56<.001	0.62<.001	—
CHAMPS, moderate calories	0.54<.001	0.40<.001	0.44<.001
CHAMPS, total calories	0.48<.001	0.33<.001	0.35<.001

RAPA indicates Rapid Assessment of Physical Activity; BRFSS, Behavioral Risk Factor Surveillance System; PACE, Patient-centered Assessment and Counseling for Exercise; CHAMPS, Community Healthy Activities Model Program for Seniors.

**Table 2:** Sensitivity, Specificity, and Predictive Value of the RAPA, BRFSS, and PACE Physical Activity Measures Compared With CHAMPS Physical Activity Level,^a^  Strength, and Flexibility^b^

**Activity Measure^c^**	**Sensitivity, %**	**Specificity, %**	**Positive Predictive Value, %**	**Negative Predictive Value, %**
RAPA	81	69	77	75
BRFSS	70	73	78	65
PACE	75	63	71	67
RAPA flexibility	85	62	87	58
RAPA strength	89	84	88	86

RAPA indicates Rapid Assessment of Physical Activity; BRFSS, Behavioral Risk Factor Surveillance System; PACE, Patient-centered Assessment and Counseling for Exercise; CHAMPS, Community Healthy Activities Model Program for Seniors.

a Defined as total of 5 days per week or more *and* total of 3 or more hours per week of all moderate-intensity physical activities (metabolic equivalent values [METs] 3.0–4.9) *or* total of 3 days per week or more *and* total of 1 or more hours per week of all vigorous-intensity physical activities (METs ≥ 5.0).

b Strength and flexibility assessed only in the CHAMPS and RAPA.

c N = 115 for all comparisons except strength (n = 113).

**Table 3:** Assessment of Mean Caloric Expenditure on the CHAMPS by Physical Activity (PA) Status on the RAPA, BRFSS, and PACE

**Activity Measure**	**No.**	**Mean Caloric Expenditure per Week (95% CI)**	*** t *Test**	***P* Value**
**RAPA **				
Inadequate PA	48	807 (462-1151)	4.81	<.001
Meets PA standard^a^	67	2243 (1755-2731)		
**BRFSS**				
Inadequate PA	51	1149 (749-1550)	3.25	.001
Meets PA standard^a^	53	2304 (1715-2894)		
**PACE**				
Inadequate PA	45	927 (585-1269)	4.02	<.001
Meets PA standard^a^	62	2217 (1673-2762)		

CHAMPS indicates Community Healthy Activities Model Program for Seniors; RAPA, Rapid Assessment of Physical Activity; BRFSS, Behavioral Risk Factor Surveillance System; PACE, Patient-centered Assessment and Counseling for Exercise; CI, confidence interval.

a Meets PA standard indicates that the individuals engaged in a sufficient amount of physical activity weekly to meet the recommendation of the Centers for Disease Control and Prevention.
